# Dietary Polyphenols in Cancer Chemoprevention: Implications in Pancreatic Cancer

**DOI:** 10.3390/antiox9080651

**Published:** 2020-07-23

**Authors:** Anita Thyagarajan, Andrew S. Forino, Raymond L. Konger, Ravi P. Sahu

**Affiliations:** 1 Department of Pharmacology and Toxicology, Boonshoft School of medicine Wright State University, Dayton, OH 45435, USA; 2 Department of Anatomy and Physiology, Boonshoft School of medicine Wright State University, Dayton, OH 45435, USA; forino.2@wright.edu; 3 Department of Pathology and Laboratory Medicine, Indiana University School of Medicine, Indianapolis, IN 46202, USA; rkonger@iupui.edu

**Keywords:** dietary polyphenols, antioxidants, reactive oxygen species, cellular signaling pathways, pancreatic cancer chemoprevention, chemotherapeutic agents

## Abstract

Naturally occurring dietary agents present in a wide variety of plant products, are rich sources of phytochemicals possessing medicinal properties, and thus, have been used in folk medicine for ages to treat various ailments. The beneficial effects of such dietary components are frequently attributed to their anti-inflammatory and antioxidant properties, particularly in regards to their antineoplastic activities. As many tumor types exhibit greater oxidative stress levels that are implicated in favoring autonomous cell growth activation, most chemotherapeutic agents can also enhance tumoral oxidative stress levels in part via generating reactive oxygen species (ROS). While ROS-mediated imbalance of the cellular redox potential can provide novel drug targets, as a consequence, this ROS-mediated excessive damage to cellular functions, including oncogenic mutagenesis, has also been implicated in inducing chemoresistance. This remains one of the major challenges in the treatment and management of human malignancies. Antioxidant-enriched natural compounds offer one of the promising approaches in mitigating some of the underlying mechanisms involved in tumorigenesis and metastasis, and therefore, have been extensively explored in cancer chemoprevention. Among various groups of dietary phytochemicals, polyphenols have been extensively explored for their underlying chemopreventive mechanisms in other cancer models. Thus, the current review highlights the significance and mechanisms of some of the highly studied polyphenolic compounds, with greater emphasis on pancreatic cancer chemoprevention.

## 1. Introduction

Natural dietary polyphenolic compounds are widely distributed in various plant sources and have been explored against several health-related ailments and their associated symptoms, due to their medicinal properties [1,2,3,4,5,6]. Polyphenols constitute a large family of phytochemicals, present in a wide variety of fruits, vegetables, flowers, and leaves [7,8,9,10,11]. Epidemiological studies also support the fact that consumption of a natural polyphenol-enriched diet could reduce the incidence or lower the risk of pathophysiologies including cardiovascular diseases [12,13,14,15,16]. However, other reports have documented contrasting or inconsistent evidence for polyphenols intake against cardiovascular diseases and cancer [17,18,19,20]. This indicates the need for conducting further randomized controlled trials and observational or behavioral studies in human subjects with such disease conditions [17,18,19,20]. Nevertheless, experimental studies have characterized that polyphenolic derivatives, including quercetin, resveratrol, etc., possess several medicinal properties, including anti-inflammatory and antioxidant activities, and therefore, have been extensively explored for cancer chemoprevention in various cancer models including pancreatic cancer [7,8,9,10,11,21].

It is well established that reactive oxygen species (ROS) attribute several modifying roles in various inflammatory and chronic diseases including cancer [22,23,24]. Under normal circumstances, the redox status of the cells is maintained by homeostasis between ROS production, and its sequestration by antioxidants [22]. While ROS generation is essential to host innate immune responses against extracellular pathogens, including bacterial and viral infections, its exacerbated production causes an imbalance in the cellular redox potential, leading to alterations in the signaling cascades [25,26,27]. Depending upon the type/nature of pro-oxidative stressors, or the changes in the autonomous cellular conditions along with the cell types, the perturbation in ROS generation of any origin (i.e., governed by various cellular compartments) have been linked with neocarcinogenesis [28,29,30,31,32]. Importantly, to overcome or mitigate such ROS-mediated events, multiple cellular antioxidant enzymes and redox proteins, including superoxide dismutase and thioredoxin, have been shown to serve as the crucial counteracting antioxidant defense systems [30,33]. As cancer cells, particularly the tumor microenvironment, exhibit higher basal levels of oxidative stress compared to the normal cells, increased levels of antioxidant defenses have been observed to circumvent ROS-mediated tumor cell damage [30,34,35]. Although most ROS-generating therapeutic agents have been documented to act as prooxidant redox modifiers and designed to target the impaired or upregulated redox machinery of tumor cells, such tumor-eradicating mechanisms have also been hypothesized and demonstrated to induce chemoresistance [36,37].

Despite recent advances in treatment modalities, including immunotherapy approaches, most chemotherapeutic agents are still the mainstay to treat a variety of cancers either alone, or in combination with other drugs (e.g., repurposing drugs, targeted therapy) [38,39,40]. However, mutations/aberrations in the cellular signaling pathways that mediate chemotherapy-ROS-induced tumor resistance result in either reduced treatment efficacy or tumor relapse after an initial anti-tumor response [41,42]. Therefore, most chemotherapeutic drugs are not considered good treatment options as a single agent for advanced-stage/metastatic cancers. As these adverse events pose one of the critical ongoing challenges in cancer treatment, several new therapeutic strategies are being considered and implemented. Natural dietary polyphenols have been examined to explore their synergy with therapeutic agents in preclinical experimental models. Given that the underlying chemopreventive mechanisms of polyphenolic compounds have been reviewed in other cancer models, the current review highlights the significance of some of the highly explored polyphenols in pancreatic cancer chemoprevention.

## 2. Polyphenol-Enriched Dietary Compounds in Pancreatic Cancer Chemoprevention

**Figure 1:**
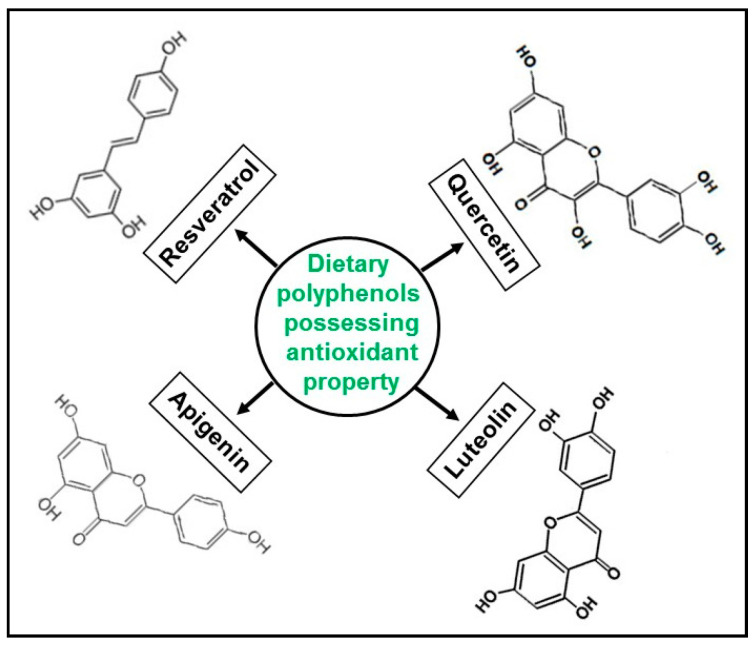
Structural representations of polyphenols.

Since the focus of this review is to highlight the significance of naturally occurring dietary polyphenols (Figure 1), given that they have not only been extensively explored for in-depth mechanistic insights, but also widely available for consumption, we excluded studies on synthetic derivatives or modified compounds of such polyphenols.

## 3. Quercetin

**Figure 2:**
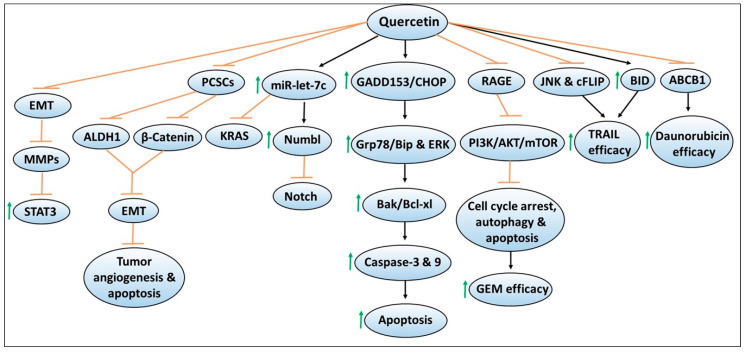
Mechanisms of quercetin-mediated chemopreventive effects as well as augmenting the sensitivity of chemotherapeutic agents against pancreatic cancer. The sign 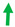 denotes increase or activation and 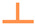 denotes inhibition or suppression.

The flavonoid quercetin (i.e., quercetin glucosides in its natural form) has been widely explored for its antineoplastic activities in various cancer models, including pancreatic cancer [43,44,45,46,47,48,49,50]. Evidence supporting its chemopreventive properties includes studies demonstrating that treatment of quercetin decreased the viability or proliferation of in vitro pancreatic cancer cell lines such as PANC-1, BxPC-3, and MiaPaCa-2 in a dose- and/or time-dependent manner [51,52]. When combined with the standard chemotherapeutic agents such as 5-fluorouracil [5-FU] or gemcitabine, growth suppression of pancreatic cancer cell lines were either found to be enhanced or relatively unchanged compared to monotherapy, indicating that quercetin may sensitize chemotherapy efficacy depending upon the cell lines used [51,52]. Figure 2 depicts mechanisms involved in quercetin-mediated chemoprevention, including its ability to enhance the sensitivity or efficacy of chemotherapeutic effects. Notably, changes in the cellular morphological features such as cell condensation, nuclear fragmentation, impaired mitochondrial membrane potential, intracellular Ca^2+^ accumulation, and cell cycle arrest have been noted in quercetin-exposed pancreatic cancer cell lines. These cellular modifications have been shown to induce apoptosis, measured by increased caspase-3 or 9 activity, or annexin V and propidium iodide staining by flow cytometry, or increased expression of the pro-apoptotic protein Bak or decreased expression of anti-apoptotic protein Bcl-xl assessed by western blotting [51,52]. Mechanistically, induction of endoplasmic reticulum (ER) stress pathways such as higher protein expression of GADD153/CHOP causing increased Grp78/Bip protein, as well as ERK activation were observed by quercetin treatment [52]. In another report, oral administration of quercetin has been found to significantly suppress the growth of orthotopically implanted pancreatic tumor xenografts compared to non-treated mice [53]. However, no additive effects were observed by quercetin in enhancing gemcitabine efficacy [53]. Quercetin-mediated inhibition of pancreatic tumor growth was accompanied by reduced tumoral BrdU, and TUNEL staining, which are the suggestive markers of decreased proliferation and increased apoptosis of tumor cells [53]. Overall, these findings indicated the promising chemopreventive effects of quercetin in in vitro and in vivo pancreatic cancer models.

An active area of research in preclinical models is to determine whether the bioavailability of orally or intraperitoneally administered quercetin results in sufficient systemic concentrations that could result in a sustained anti-tumoral activity. In the intestine, quercetin undergoes hydrolysis by lactase phlorizin hydrolase, resulting in subsequent diffusion as quercetin aglycones are actively transported by sodium-dependent glucose transporter, and then, subsequent deglycosylation by cytosolic β-glucosidase [54]. Studies by Zhang et al. evaluated the uptake of quercetin in MiaPaCa-2 pancreatic cancer cells in vitro, as well as its in vivo distribution in murine pancreatic tumor xenografts, plasma, lung, liver and pancreas [55]. The authors demonstrated that following treatment with 30 µM quercetin, it quickly accumulates in MiaPaCa-2 cells at nmol/mg protein concentration within 30 min, but its level decreases gradually in a time-dependent manner [55]. Similarly, nude mice harboring orthotopic MiaPaCa-2 tumor xenografts, and fed with 0.2% or 1% or 5% quercetin in an AIN93G-based diet for a period of 6–8 weeks, have found to exhibit varying levels of quercetin and its metabolite isorhamnetin in plasma and tumors, as well as in liver, lung and pancreas, which indicates its effective absorption [55]. While cotreatment of gemcitabine (120 mg/kg per mouse, i.p.) with quercetin (1%) has been found to result in significantly decreased growth of pancreatic tumors, gemcitabine reduced quercetin absorption in the circulatory system and liver, but not in other tissues [55]. These studies suggested that quercetin gets readily metabolized and bioavailable to exert a systemic anti-tumoral effect as well as augments chemotherapy efficacy. However, further studies are warranted to verify whether or not quercetin can also enhance the metabolism and absorption of chemotherapy agents.

Several cellular mechanisms by which quercetin treatment inhibits the growth of pancreatic cancer cell lines in in vitro and in vivo models, have been identified. The authors have also explored the cellular model systems to test the sensitivity or resistance to agents, to further define the translational significance of quercetin to be used in combination treatment approaches for pancreatic cancer. In one such study, Borska et al. determined the effects of quercetin on the daunorubicin sensitive EPP85-181P cell line, as well as the daunorubicin resistant EPP85-181RDB cell line [56]. Quercetin exposure was found to reduce cell proliferation and increase apoptosis in both the cell lines in a dose-dependent manner. Importantly, quercetin combined with daunorubicin induced synergistic effects in daunorubicin-sensitive cells, and also, sensitized the daunorubicin-resistant cells to daunorubicin cytotoxic effects [56]. Another report by the same group later identified the role of P-glycoprotein (*ABCB1*), one of the ABC transporters reported to induce chemoresistance via its ability to expel drugs outside of cells [57]. In this study, the authors determined P-glycoprotein was involved in the ability of quercetin to promote daunorubicin-induced cytotoxicity in both daunorubicin-resistant EPP85-181RDB and daunorubicin sensitive EPP85-181P cell lines [57]. They demonstrated that P-glycoprotein expression was 51-fold higher in the resistant cells compared to the sensitive cells and that quercetin treatment decreased P-glycoprotein expression by 35% in the resistant cell line and by 67% in the sensitive cell line [57]. These findings indicated that quercetin possesses the ability to overcome tumor resistance mechanisms, and thus, could be used to enhance the efficacy of chemotherapeutic agents.

### 3.1. Effects of Quercetin on Pancreatic Cancer Stem Cells and EMT

Targeting pancreatic cancer stem or stem-like cells (PCSCs) has been explored as a promising approach to regulate the aggressiveness and metastatic ability of tumor cells [58,59]. In this regard, quercetin treatment has been shown to reduce PCSCs’ ability to induce in vitro spheroid/colony formation, or in vivo growth of stem-cell enriched tumor xenografts [60]. These effects were mediated via mechanisms involving diminishing ALDH1 activity, preventing epithelial to mesenchymal transition (EMT), reducing tumor cell angiogenesis, induction of apoptosis, as well as decreased expression of CSCs markers [60]. Importantly, these quercetin-mediated effects were enhanced by the combination of another dietary isothiocyanate compound, sulforaphane. Overall, the data suggested that additive or synergistic chemopreventive effects could be achieved with the use of more than one natural compound possessing similar mechanisms of action.

Another study utilized a culture sphere model of PCSCs along with their respective parental cells to determine the interaction and underlying mechanisms of quercetin-mediated inhibition of PCSCs [61]. The studies identified increased expression of β-catenin in the parental cells compared to PCSCs. In contrast, PCSCs exhibited increased resistance to gemcitabine compared to parental cells [61]. Importantly, quercetin treatment resulted in decreased proliferation, invasion, and sphere-forming capacity of PCSCs, and also, reduced the expression of CSCs markers [61]. Notably, these effects were found to be associated with the alterations in β-catenin expression, and that quercetin combined with gemcitabine not only reduced the growth of PCSCs but also their inherent chemoresistance to gemcitabine [61]. These findings indicated the potential of targeting β-catenin to improve gemcitabine efficacy against pancreatic cancer.

In another report, quercetin treatment has been shown to suppress the growth of PANC-1 and PATU-8688 pancreatic cancer cell lines via inhibiting EMT and decreasing the secretion of matrix metalloproteinase (MMP) [62]. These quercetin-induced effects were found to be mediated via mechanisms involving the reduced activation and activity of signal transducer and activator of transcription 3 (STAT3) [62]. To verify this mechanism, the authors demonstrated that quercetin blocked IL-6-induced STAT3 activation, EMT and MMP secretion that resulted in increased migration and invasion of pancreatic cancer cell lines [62]. These findings also indicate that inhibition of such pathways with resveratrol can result in higher chemopreventive effects in pancreatic cancer.

### 3.2. Quercetin and MicroRNA

Given that the altered expression of microRNAs (miRs) have been documented in clinical samples, they have been extensively studied for their roles in modulating cancer growth and the efficacy of therapeutic agents in tumor models that include pancreatic cancer [63,64,65]. Nwaeburu et al. performed miRs profiling in PDA cells with or without quercetin treatment and identified miR let-7c as one of the highly upregulated miRs following quercetin treatment [66]. The authors also identified NUMB like endocytic adaptor protein (*NUMBL)*, a Notch inhibitor as a target of let-7c. Similar to quercetin, transfection of let-7c mimics induced a wildtype construct of *NUMBL* 3′UTR, its mRNA and protein expression, but inhibited Notch protein expression [66]. The in vitro transfection or in vivo intravenous injection of let-7c resulted in the inhibition of colony formation, wound healing, and cell proliferation, or decreased mass of PDA tumor in the fertilized check egg model [66]. These findings suggested that quercetin-induced let-7c serves as a novel mechanism for suppressing pancreatic tumor growth [66]. This idea is further supported by increased expression of miR-let7-a by quercetin and other dietary agents in pancreatic cancer models but not in normal cells, and that miR-let7-a induction was found to be associated with K-ras inhibition [67]. The combination of quercetin with sulforaphane or green tea catechins complemented each other and resulted in enhanced inhibition of PDA progression. This effect was found to be mediated via miR-let7-a induction and K-ras inhibition [67]. Given these findings, one can extrapolate that miR-let7-a expression could be used to assess the efficacy of quercetin against pancreatic cancer.

Importantly, bioinformatics tools and database analysis have also been explored to predict the new mechanisms and targets of quercetin in cancer models, including pancreatic cancer [68]. Of various targets, CD36 and thrombospondin-1 were identified to be targeted by quercetin in pancreatic cancer [68].

### 3.3. Quercetin Effects on Chemotherapy Efficacy

Given the improved effectiveness of quercetin when combined with other dietary agents [60,67], quercetin has been tested for its ability to enhance the sensitivity of chemotherapeutic agents. In one such study, Kim et al. have found that quercetin combined with tumor necrosis factor-related apoptosis-inducing ligand (TRAIL), an anticancer agent, augmented TRAIL-induced apoptotic response in TRAIL-resistant pancreatic cancer cells [69]. These effects were mediated via mechanisms involving c-Jun N-terminal kinase (JNK), FLICE-like inhibitory protein (cFLIP), and BH3-only pro-apoptotic protein BID [69]. Additional studies demonstrated that the activation of JNK, overexpression of cFLIP, or BID knockdown rescued pancreatic cancer cells to TRAIL and quercetin-induced apoptosis [69]. As the receptor for advanced glycation end products (RAGE) expression has been implicated in inducing gemcitabine resistance, a study led by Lan et al. tested if quercetin can enhance gemcitabine sensitivity via targeting RAGE in gemcitabine-resistant pancreatic cancer cells [70]. The authors demonstrated that quercetin treatment downregulated RAGE expression, which resulted in cell cycle arrest, autophagy and apoptosis induction. These effects were mediated via the inhibition of the PI3K/AKT/mTOR pathway, which resulted in enhanced gemcitabine sensitivity in gemcitabine-resistant pancreatic cancer cells [70]. Overall, these findings suggested that quercetin due to its ability to target a wide array of signaling pathways could be used as a promising combination approach to enhance the efficacy of chemotherapy agents against pancreatic cancer.

## 4. Resveratrol

**Figure 3:**
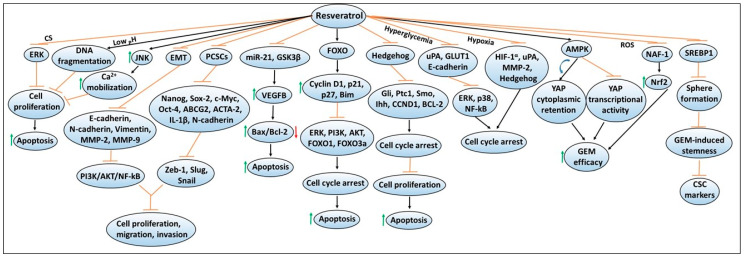
Resveratrol-mediated mechanisms involved in exerting chemopreventive effects and enhancing chemotherapy efficacy against pancreatic cancer. The sign 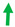 denotes increase or activation, 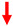 decrease expression or downregulation, 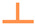 inhibition or suppression, and 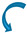 induction.

Similar to quercetin, another polyphenol resveratrol (trans-3,5,4′-trihydroxystilbene) is widely distributed in various fruits and has been characterized due to its powerful antioxidant property in various disease models, including cancers [71,72,73,74,75,76]. Importantly, resveratrol has been extensively studied to understand the mechanisms of its anti-proliferative and anti-carcinogenic properties that induce programmed cell death (i.e., apoptosis) [77,78,79]. Figure 3 depicts mechanisms involved in resveratrol-mediated chemoprevention, including its ability to enhance the sensitivity or efficacy of gemcitabine chemotherapy. A study done by Cui et al. has shown that resveratrol treatment inhibited the proliferation of pancreatic cancer cell lines, PANC-1, BxPC-3, and AsPC-1 in a dose- and time-dependent manner [80]. These findings along with other studies have found that resveratrol was able to reduce colony formation or promote apoptosis by increasing the expression of pro-apoptotic proteins such as Bax and caspase-3 while inhibiting the expression of anti-apoptotic proteins such as Bcl-2, and Bcl-xL, as well as metalloenzymes such as leukotriene A_4_ hydrolase (LTA_4_H) [80,81]. Another study has shown that resveratrol’s pro-apoptotic activity includes the involvement of the mitochondrial pathway [82]. The experimental findings revealed that resveratrol treatment resulted in depolarization of mitochondria membrane potential (consistent with mitochondrial dysfunction), leading to increased apoptosis as measured by Annexin V/PI staining [82]. Similar pro-apoptotic mechanisms for resveratrol have been identified in other tumor models such as lung, prostate, and colorectal cancers [83,84,85]. In another study, Garcia-Sanchez et al. have shown that resveratrol stimulates intracellular Ca^2+^ mobilization by inducing JNK activation, and this resulted in decreased viability of AR42J pancreatic cancer cells [86]. In addition, resveratrol-induced cell cycle arrest or apoptosis in PANC-1, MIAPaCa-2, Hs766T and AsPC-1 cell lines were found to be mediated via the activation of forkhead (FOXO) family of transcription factors, and upregulation of FOXO target genes such as cyclin D1, p21/CIP1, p27/KIP1 and Bim, as well as cleaved caspase-3 [87]. These changes also resulted in reduced phosphorylation of ERK, PI3K, AKT, FOXO1 and FOXO3a [87]. Overall, these findings indicated that there are involvements of a wide array of cellular signaling pathways in mediating chemopreventive effects of resveratrol in pancreatic cancer.

### 4.1. Other Cellular Targets of Resveratrol

In addition, the inhibition of the hedgehog signaling pathway, as well as decreased protein levels of other family members or transcript expression of downstream target genes (i.e., Gli1, Ptc1, Smo, *CCND1* and *BCL-2*) were observed by resveratrol treatment [88]. These mechanistic changes resulted in G0/G1 cell cycle arrest, and increased apoptosis in PANC-1, AsPC-1 or BxPC-3 pancreatic cancer cell lines [88]. Moreover, resveratrol-mediated a dose- and time-dependent decrease in cellular proliferation, and induction of apoptosis, in the Mia PaCa-2 pancreatic cancer cell line [89]. This was found to be mediated via the inhibition of the hedgehog signaling pathway, and downregulation of protein and mRNA expression of Ihh, Ptch, and Smo genes [89]. Moreover, resveratrol has also been found to downregulate the mRNA expression of miR-21, and protein expression of glycogen synthase kinase 3 beta (GSK3β), while upregulation of the protein and mRNA levels of the growth factor, vascular endothelial growth factor B (VEGF-B), was observed [90,91]. These mechanistic changes induced increased expression of the pro-apoptotic Bax protein and decreased expression of the anti-apoptotic Bcl-2 protein, which resulted in enhanced apoptosis in PANC-1, CFPAC-1, MiAPaCa-2 and Capan-2 pancreatic cancer cell lines [90,91]. Importantly, overexpression of miR-21 has been found to reverse Bcl-2 protein downregulation, and apoptosis induction by resveratrol [90]. Similarly, VEGF-B silencing via the siRNA approach has been reported to upregulate GSK3β protein expression and increase the rate of apoptosis [91]. Importantly, the resveratrol combination resulted in a significantly higher apoptotic rate compared to VEGF-B silencing or resveratrol alone treatments [91]. These findings also indicate that inhibitors of these cellular targets could be explored with resveratrol to achieve higher chemopreventive effects in pancreatic cancer.

### 4.2. Effects of Extracellular Environments on Resveratrol’s Chemopreventive Responses

The generation of ROS along with extracellular environmental factors have been studied in various cancer models due to their ability to affect multiple cellular signaling pathways that can modify the functionality of critical pro- and anti-apoptotic genes [92,93,94]. These morphological and physiological changes not only help support cancer cells’ survival but also favor their invasive capabilities [94,95,96]. A study by Shamim et al. explored resveratrol’s effects in low pH environments for tumors that prefer specific acidic environments [96]. The studies demonstrated that the ability of resveratrol to inhibit the growth and to induce internucleosomal DNA fragmentation-induced apoptosis was enhanced at lower pH in Capan-2 and Panc-28 pancreatic cancer cell lines [96]. Subsequently, other studies have also examined resveratrol’s effects on pancreatic cancer cells under two different extracellular environmental (i.e., hyperglycemic and hypoxic) conditions [97,98]. One study involving the extracellular hyperglycemic environment showed that it was able to induce cell cycle arrest in PANC-1 cell line via inhibiting urokinase plasminogen activator (uPA), E-cadherin, and glucose transporter 1 (GLUT1) expression. These changes resulted in the suppression of ERK and p38 MAPK signaling pathways, as well as the transcription factor NF-κB [97]. Another study involving the extracellular hypoxic environment revealed that resveratrol mediated cell cycle arrest in BxPC-3 and PANC-1 cell lines through its ability to suppress hypoxia-inducible factor-1 alpha (HIF-1α), uPA and MMP-2 protein expression, as well as inhibiting hypoxia-mediated activation of the hedgehog signaling pathway [98]. Since ROS produced by cigarette smoking (CS) can also increase cancer risk, a study explored the effects of resveratrol under this extracellular environment and found that resveratrol was able to suppress CS-induced increased cellular proliferation of pancreatic cancer cells via downregulating pERK expression [99]. Overall, these studies suggested that resveratrol was able to mitigate extracellular environment-induced growth-enhancing effects on pancreatic cancer cells.

### 4.3. Effects of Resveratrol on Pancreatic Cancer Stem Cells and EMT

Resveratrol has also been examined for its ability to regulate EMT, which is involved in tissue repair but has also been implicated in contributing to the progression of cancer [100,101,102]. Cancer cells that acquire mesenchymal features also develop the ability to escape from the primary tumor site and metastasize to other organs [103]. Thus, the regulation of this mechanism has become a focal point in cancer research. In an investigation of how resveratrol alters EMT regulation using BxPC-3 and PANC-1 pancreatic cancer cell lines, it was found that resveratrol inhibits the cellular proliferation, migration, and invasion in a dose-dependent manner via targeting the expression of EMT-related genes such as E-cadherin, N-cadherin, vimentin, MMP-2 and MMP-9, as well as PI3K/Akt/NF-κB signaling pathways [104]. Notably, studies have also been directed to define resveratrol’s anti-cancerous characteristics with EMT within pancreatic cancer stem cells (PCSCs) possessing stem cell ability. One study found that resveratrol can inhibit PCSCs characteristics by inhibiting pluripotency maintaining factors such as Nanog, Sox-2, c-Myc and Oct-4, as well as the drug resistance gene, *ABCG2*, in CSCs possessing CD133 + CD44 + CD24 + ESA + phenotype. These findings were found to be associated with the suppression of migration and invasion, as well as EMT markers such as Zeb-1, Slug, and Snail [105]. Another study reported that CD133 + PCSCs exhibit a significant reduction in ACTA-2, IL-1β and N-cadherin immunoreactivities by resveratrol treatment [106]. These findings implicated that resveratrol could be used to prevent EMT in pancreatic cancer cells.

### 4.4. Resveratrol Effects on Chemotherapy Efficacy

As combination chemotherapy approaches are being considered as promising strategies for cancer treatment [107,108], several studies have explored the effectiveness of resveratrol in combination with chemotherapeutic agents or other natural compounds. One possible reason for this is that while ongoing therapeutic regimens aid marginal survival benefits, patients often experience tumor relapse, and their tumors become more resistant to the identical treatment options [109]. Given that resveratrol alone can decrease cellular proliferation by inducing G1 cell cycle arrest, cyclin D1 downregulation, and inactivation of AKT-GSK3β and ERK1/2 signaling [110], other studies have shown that resveratrol combined with gemcitabine chemotherapy is promising as a potential therapeutic option against pancreatic cancer [111,112,113,114]. Jiang et al. have demonstrated that resveratrol treatment enhanced the sensitivity of pancreatic cancer cells to gemcitabine via inducing AMP-activated protein kinase (AMPK) signaling, resulting in the induction of yes-associated protein (YAP) cytoplasmic retention, and the inhibition of YAP transcriptional activity [112]. Another study found that resveratrol treatment suppressed the expression of nutrient-deprivation autophagy factor-1 (NAF-1) in pancreatic cancer cells by inducing ROS accumulation. This led to the activation of nuclear factor erythroid 2-related factor 2 (Nrf2) signaling, and that these mechanistic modifications resulted in improved sensitivity of pancreatic cancer cells to gemcitabine [113]. Not only can resveratrol enhance chemotherapeutic response, but a recent study done by Zhou et al. showed that it can also reverse the stemness of pancreatic cancer cells induced by gemcitabine [114]. In this study, resveratrol treatment was found to enhance sensitivity to gemcitabine by inhibiting lipid synthesis via SREBP1, which limited the sphere formation ability and suppressed gemcitabine-induced stemness, as well as the expression of CSC markers [114]. Overall, these findings indicated resveratrol’s response in augmenting chemotherapy efficacy against pancreatic cancer due to its diverse targetability of signaling pathways.

## 5. Apigenin

**Figure 4:**
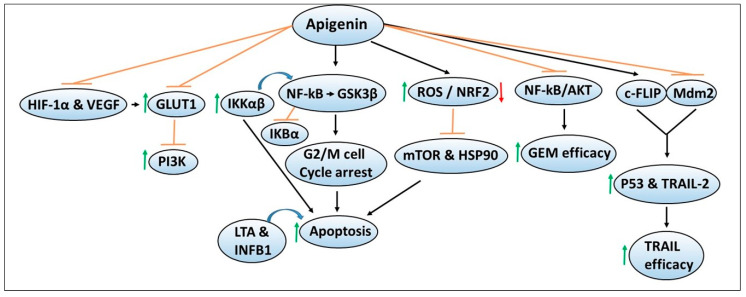
Mechanisms of apigenin-mediated chemopreventive effects as well as augmenting the sensitivity of chemotherapeutic agents against pancreatic cancer. The sign 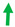 denotes increase or activation, 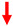 decrease expression or downregulation, 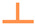 inhibition or suppression, and 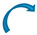 induction.

Malignant cells require nutrients such as glucose and amino acids to support their sustained growth, and in their absence, tumor cells scavenge extracellular proteins such as albumin for their survival [115,116]. To transport the specific nutrients, cancer cells acquire transport mechanisms, such as glucose transporters for the transport of glucose, etc., [117,118]. Therefore, agents that can inhibit such transporters or their metabolism are being explored as promising therapeutic agents for cancer intervention. Figure 4 depicts the chemoprevention mechanisms of apigenin and its ability to enhance the sensitivity or efficacy of chemotherapeutic agents. In one study, the effect of apigenin was evaluated on GLUT1 expression, and the mechanisms regulating its expression were determined in CD18 and S2-013 human pancreatic cancer cell lines [119]. The studies demonstrated that apigenin inhibited GLUT1 expression and glucose uptake in a time- and dose-dependent manner, similar to that observed by PI3K inhibitors, suggesting that the apigenin effects were mediated via the inhibition of PI3K signaling [119]. Studies by the same group later reported that hypoxic conditions can also upregulate the protein and mRNA expression of GLUT1, as well as hypoxia-related HIF-1α and VEGF, which were reversed by apigenin in CD18 and S2-013 pancreatic cancer cell lines [120]. These findings could offer additional mechanisms that apigenin can target in both normoxic and hypoxic conditions for pancreatic cancer chemoprevention.

In another report, the authors determined the interaction of apigenin with GSK3β [121]. Molecular docking studies predicted that apigenin binds within the GSK3β cavity with low interaction energies, which leads to the inhibition of its enzymatic activity [121]. Similar results were observed with other flavonoids such as quercetin and luteolin, indicating their potential in targeting GSK3β signaling to suppress pancreatic cancer. Apigenin has also been shown to induce G2/M cell cycle arrest and apoptosis in BxPC-3 and PANC-1 pancreatic cancer cell lines via targeting GSK3β/NF-kB signaling cascade [122]. Increased expression of cytokines (including the IL17 family, LTA and INFB1) was correlated with increased apigenin-induced apoptosis [122]. Given the critical roles of NF-kB in cancer progression, studies by Wu et al. determined the regulatory mechanism of NF-kB in the context of apigenin [123]. Apigenin treatment decreased cell survival and increased apoptosis of AsPC-1, PANC-1 and MiaPaCa-2 pancreatic cancer cell lines through the inhibition of tumor necrosis factor-alpha (TNFα)-induced DNA binding (p65 and p50 subunits), resulting in reduced transcriptional activities of NF-kB [123]. Decreased NF-kB activity was associated with IkBα degradation, reduced expression, and activation of upstream IKK (α and β subunits), and reduced NF-kB nuclear translocation [123]. Notably, the NF-kB inhibitor Bay11-7082 enhanced the chemosensitivity of apigenin, and IKKβ overexpression attenuated apigenin-induced decreased cell survival. Importantly, suppression of AsPC-1 tumor growth by apigenin was also correlated with decreased protein expression and phosphorylation of IKKα/β and increased apoptosis [123]. These findings indicated that apigenin targets IKK-mediated NF-kB activation to inhibit pancreatic cancer growth. From these studies, it can be postulated that apigenin could be explored in combination with the pharmacological inhibitors of these pathways/targets in widely used transgenic experimental pancreatic cancer models.

### 5.1. Other Cellular Targets of Apigenin

Multiple studies have also defined other mechanisms of apigenin-mediated chemopreventive effects in pancreatic cancer models. In one study, apigenin-induced growth suppression and increased apoptosis were found to be mediated by post-translational modification, nuclear translocation, and DNA binding of p53, as well as p21 and PUMA upregulation in BxPC-3 and MiaPaCa-2 cell lines with mutated p53 expression [124]. However, further studies demonstrated p53 DNA binding activity and transcriptional activity were not necessary for the observed ability of apigenin to suppress cell growth or increase apoptosis. Additional confirmatory studies suggested that the in vitro and in vivo effects of apigenin could be mediated via the augmentation of transcription-independent p53 functions, despite its deactivating mutations [124]. Importantly, another group demonstrated differential cytotoxic effects of apigenin in PANC-1 and PaCa44 pancreatic cancer cell lines, which harbor different p53 mutations [125]. The PANC-1 cell line exhibited greater apigenin cytotoxicity compared to the PaCa44 cell line, which was dependent on increased induction of intracellular ROS/decreased antioxidant defenses, mutant (mut) p53 reduction, as well as inhibition of mTOR and heat shock protein 90 (HSP90) [125]. These findings suggested that an interplay between mTOR-HSP90-mut p53-p62-NRF2 mediates apigenin chemoresistance in p53 mutated pancreatic cancer.

### 5.2. Apigenin Effects on Chemotherapy Efficacy

In addition to exerting cancer chemoprevention effects, apigenin has also been tested for its efficacy in enhancing the efficacy of anticancer agents. Notably, the combination of apigenin with gemcitabine has been shown to cause enhanced cytotoxic effects in in vitro and in vivo pancreatic cancer models compared to apigenin or gemcitabine alone [126]. These effects were found to be mediated due to the inhibition of NF-kB and Akt activation [126]. Similarly, another study also reported that apigenin and gemcitabine combination resulted in higher cytotoxic effects than individual treatment [127]. Mechanistically, the enhanced effects of combination therapy were found to be mediated via S and G2/M cell cycle arrest, and inhibition of Akt signaling leading to increased apoptosis in CD18 and AsPC-1 pancreatic cancer cell lines [127]. Another study examined whether apigenin and other related flavones could overcome resistance to apoptosis in response to the treatment with TRAIL [128]. This was done using the TRAIL-resistant human T cell leukemia virus type 1 (HTLV-1)-associated adult T cell leukemia (ATL) cellular model [128]. Mechanistic studies demonstrated that apigenin downregulates the protein expression of c-FLIP via inhibiting Mdm2 that antagonizes p53. This upregulated p53 activity as well as the expression of its downstream target TRAIL-receptor 2, which in turn augmented TRAIL-induced apoptosis [128]. Similar effects were observed in multiple cancer cell lines, including Capan-1 pancreatic cancer cells, indicating the potential use of natural flavone compounds such as apigenin as an adjuvant to improve the efficacy of anticancer agents such as TRAIL.

## 6. Luteolin

**Figure 5:**
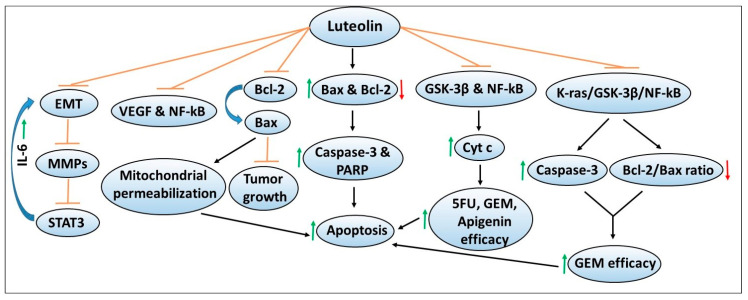
Cellular signaling pathways targeted by luteolin in mediating the chemopreventive effects as well as augmenting the efficacy of chemotherapeutic agents against pancreatic cancer. The sign 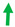 denotes increase or activation, 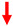 decrease expression or downregulation, 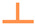 inhibition or suppression, and 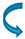 or 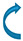 induction.

Another dietary polyphenol flavonoid that has been extensively studied in cancer models including pancreatic cancer is luteolin. Multiple studies have explored the role and chemopreventive mechanisms of luteolin using various in vitro and in vivo models of pancreatic cancer. Figure 5 depicts the chemoprevention mechanisms of apigenin and its ability to enhance the sensitivity or efficacy of chemotherapeutic agents. A study led by Cai et al. demonstrated that luteolin treatment inhibits the proliferation of PANC-1, CoLo-357 and BxPC-3 pancreatic cancer cell lines via inducing apoptosis [129]. These effects were mediated through increased caspase-3 and poly ADP-ribose polymerase (PARP) cleavage associated with increased expression of pro-apoptotic Bax, and decreased expression of anti-apoptotic Bcl-2 proteins [129]. Using the HUVEC cell line, a widely used model to study tubule formation or angiogenesis, the authors demonstrated that luteolin inhibited HUVEC cell proliferation and capillary formation via its ability to suppress the transcriptional activity of NF-kB, resulting in reduced VEGF expression and secretion [129]. These findings indicated that luteolin inhibits angiogenic as one of the mechanisms to induce apoptosis in pancreatic cancer cells.

### 6.1. Other Cellular Targets of Luteolin

Notably, increased Bcl-2 expression has been implicated in tumor progression, and the induction of chemoresistance in cancer models, as well as correlated with poor prognosis in cancer patients [130,131,132,133,134]. Luteolin treatment has been found to induce apoptosis in Bcl-2 overexpressing SW1990 pancreatic cancer cells via inhibiting Bcl-2 expression in a dose-dependent manner [135]. Cellular thermal shift and competitive binding assays revealed that luteolin directly binds to Bcl-2 and displaces BAX from its hydrophobic cleft, resulting in mitochondrial permeabilization leading to apoptosis [135]. Similarly, luteolin treatment resulted in a significant reduction in SW1990 tumor xenograft growth, indicating its potential in targeting Bcl-2 overexpressing pancreatic cancer [135].

Another study determined the mechanisms of luteolin in regulating EMT and cancer invasiveness using PANC-1 and SE1990 pancreatic cancer cell lines [136]. Luteolin treatment in a dose-dependent manner inhibited EMT, protein expression of MMPs (i.e., MMP2/7/9), and STAT3 signaling, as well as decreased the invasiveness of pancreatic cancer cells [136]. Treatment with IL-6, an upstream regulator of STAT3, enhanced EMT, MMP secretion and STAT3 activity in a process blocked by luteolin, indicating its chemopreventive potential [136]. Overall, these findings also suggest that pharmacological inhibitors of these pathways could be explored in combination with luteolin to achieve enhanced chemopreventive effects in pancreatic cancer models.

### 6.2. Luteolin Effects on Chemotherapy Efficacy

Given luteolin’s beneficial effects against pancreatic cancer, studies by Johnson et al. evaluated its combination with chemotherapeutic agents using an in vitro BxPC-3 pancreatic cancer cell model [137]. While concurrent treatment of luteolin in combination with either 5FU or gemcitabine resulted in relatively less cytotoxic effects, pretreatment with luteolin sensitized the cells to the growth inhibitor effects of these chemotherapeutic agents. These effects were mediated via decreased expression of GSK3β and NF-kB and increased the release of the pro-apoptotic cytochrome c protein [137]. Similar effects were observed with another flavonoid apigenin. Another study by the same group tested the effects of luteolin in combination with gemcitabine using an in vivo orthotopic model of pancreatic cancer [138]. The authors demonstrated that combination therapy with luteolin and gemcitabine resulted in a significant reduction in tumor growth compared to either the control group, or the two single-agent treatment groups [138]. Mechanistically, inhibition of the K-ras/GSK3β/NF-kB pathway by the luteolin + gemcitabine combination therapy was found to correlate with reduced tumor cell proliferation and increased apoptosis as confirmed by increased caspase-3 activation and decreased Bcl-2/Bax ratio and cytochrome c release [138]. These findings indicated that luteolin targets similar signaling pathways both in the in vitro and in vivo models, to inhibit growth or enhance chemotherapy sensitivity against pancreatic cancer.

## 7. Conclusions

Numerous studies indicate a role for antioxidant-enriched polyphenols in regulating the growth and metastasis of experimental pancreatic cancer models. While data regarding the safety and benefits of these polyphenols used during cancer treatment are largely absent, preliminary cell line and animal studies suggest a potential benefit. Importantly, the potential of these polyphenols in enhancing the efficacy of standard chemotherapeutic agents, and other natural compounds against experimental pancreatic cancer provide a rationale for their exploration in clinical settings. As large percentage of cancer patients undergoing active treatments use antioxidant-enriched compounds of all natural sources, and not all are likely to be beneficial; further preclinical and clinical studies are needed to establish the use of such polyphenols as adjuvants, to determine their optimal doses and timings for the management of cancer in combination with therapeutic regimens. Importantly, more preclinical studies determining the pharmacokinetic and pharmacodynamic profiles of these polyphenols are needed to assess the bioavailability of related compounds/metabolites. These studies would help optimize the dosing regimens of these natural polyphenols to evaluate their anti-tumor efficacy alone and in combination with therapeutic agents. Furthermore, bioinformatics studies are needed to predict the targets of these polyphenols so their pharmacological inhibitors could be tested to verify the mechanisms in cellular and preclinical studies.

## Abbreviations

Reactive oxygen species (ROS); 5-fluorouracil (5-FU); Growth arrest and DNA damage 153 (GADD153); C/EBP homologous protein (CHOP); Glucose regulated protein 78-kDa (Grp78); Binding immunoglobulin protein (Bip); Pancreatic cancer stem cell or stem-like cells (PCSCs); Aldehyde dehydrogenase 1 (ALDH1); Epithelial to mesenchymal transition (EMT); Signal transducer and activator of transcription 3 (STAT3); Interleukin 6 (IL-6); MicroRNA (miR); NUMB like endocytic adaptor protein (NUMBL); Tumor necrosis factor-related apoptosis-inducing ligand (TRAIL); c-Jun N-terminal kinase (JNK); FLICE-like inhibitory protein (cFLIP); Receptor for advanced glycation end products (RAGE); Phosphatidylinositol-3-kinase (PI3K); mammalian target of rapamycin (mTOR); leukotriene A_4_ hydrolase (LTA_4_H); Forkhead family of transcription factor (FOXO); Indian hedgehog (Ihh); Patched (Ptch); Smoothened (Smo); Glycogen synthase kinase 3 beta (GSK3β); Vascular endothelial growth factor B (VEGF-B); Glucose transporter 1 (GLUT1); Mitogen activated protein kinase (MAPK); Extracellular-signal-regulated kinase (ERK); Nuclear factor-kappa B (NF-kB); Hypoxia-inducible factor 1 alpha (HIF-1α); Urokinase plasminogen activator (uPA); Matrix metalloproteinase (MMP); AMP-activated protein kinase (AMPK); Yes-associated protein (YAP); Nutrient-deprivation autophagy factor-1 (NAF-1); Nuclear factor erythroid 2-related factor 2 (Nrf2); tumor necrosis factor alpha (TNFα); heat shock protein 90 (HSP90); poly ADP-ribose polymerase (PARP).
